# Novel Processed Form of Syndecan-1 Shed from SCC-9 Cells Plays a Role in Cell Migration

**DOI:** 10.1371/journal.pone.0043521

**Published:** 2012-08-15

**Authors:** Annelize Z. B. Aragão, Marília Belloni, Fernando M. Simabuco, Mariana R. Zanetti, Sami Yokoo, Romênia R. Domingues, Rebeca Kawahara, Bianca A. Pauletti, Anderson Gonçalves, Michelle Agostini, Edgard Graner, Ricardo D. Coletta, Jay W. Fox, Adriana F. Paes Leme

**Affiliations:** 1 Mass Spectrometry Laboratory, Brazilian Biosciences National Laboratory – CNPEM, Campinas, Brazil; 2 Piracicaba School of Dentistry, University of Campinas (UNICAMP), Piracicaba, Brazil; 3 School of Medicine, University of Virginia, Charlottesville, Virginia, United States of America; National Center for Scientific Research Demokritos, Greece

## Abstract

The extracellular milieu is comprised in part by products of cellular secretion and cell surface shedding. The presence of such molecules of the sheddome and secretome in the context of the extracellular milieu may have important clinical implications. In cancer they have been hypothesized to play a role in tumor growth and metastasis. The objective of this study was to evaluate whether the sheddome/secretome from two cell lines could be correlated with their potential for tumor development. Two epithelial cell lines, HaCaT and SCC-9, were chosen based on their differing abilities to form tumors in animal models of tumorigenesis. These cell lines when stimulated with phorbol-ester (PMA) showed different characteristics as assessed by cell migration, adhesion and higher gelatinase activity. Proteomic analysis of the media from these treated cells identified interesting, functionally relevant differences in their sheddome/secretome. Among the shed proteins, soluble syndecan-1 was found only in media from stimulated tumorigenic cells (SCC-9) and its fragments were observed in higher amount in the stimulated tumorigenic cells than stimulated non-tumorigenic cells (HaCaT). The increase in soluble syndecan-1 was associated with a decrease in membrane-bound syndecan-1 of SCC-9 cells after PMA stimuli. To support a functional role for soluble syndecan-1 fragments we demonstrated that the synthetic syndecan-1 peptide was able to induce cell migration in both cell lines. Taken together, these results suggested that PMA stimulation alters the sheddome/secretome of the tumorigenic cell line SCC-9 and one such component, the syndecan-1 peptide identified in this study, was revealed to promote migration in these epithelial cell lines.

## Introduction

Oral cancer is one of the most common malignancies worldwide and despite improvements in diagnosis and treatment, the overall survival rate for advanced patients has not been significantly improved over the last three decades [1]. Indeed, the lack of biomarkers avoids prognostic prediction or specific treatment for oral squamous cell carcinomas (OSCC), the most common presentation of oral cancer.

New approaches on clinical proteomics, such as secretome-based analysis, have been developed to identify novel biomarkers. Secretome/sheddome is a proteomic area that allows the analysis of a dynamic extracellular environment including secreted, released, degraded or shed proteins [2]–[4]. Soluble proteins in the extracellular milieu can have specific functions and can induce a variety of responses that are still not predictable, for instance, notch, E-cadherin and CD44 are known candidates for potential outside-in signal transduction [5]–[8]. These fragments can carry over conserved sequences that can regulate autocrine and paracrine targets [9].

In order to evaluate the differences between the secretome/sheddome of normal and tumorigenic cells, two epithelial cell lines, HaCaT and SCC-9, were treated with phorbol-ester (PMA). Here we showed that PMA stimulation induced distinct migration, adhesion and gelatinase activity as well as differences in the secretome/sheddome. Components in the media such as soluble and fragments of syndecan-1 were found mainly in stimulated tumorigenic cells. Syndecans are known family of cell surface proteoglycans that play regulatory roles in many biological processes, including migration, proliferation, wound healing, inflammation, angiogenesis and tumorigenesis [10], [11]. The role of syndecans in tumor progression may vary with tumor stage and type [10]. In squamous cell carcinoma, the reduction of syndecan-1 expression is correlated with the progression of carcinogenesis [12], histological grade of malignancy [13], tumor size and the mode of invasion [14]. Furthermore, we also demonstrated evidence that the fragment of syndecan-1 identified was able to induce cell migration.

## Results

### Analysis of secretome/sheddome in tumorigenic and non-tumorigenic cells

#### Secretome/sheddome composition is distinct in tumorigenic and non-tumorigenic cells

**Figure 1:**
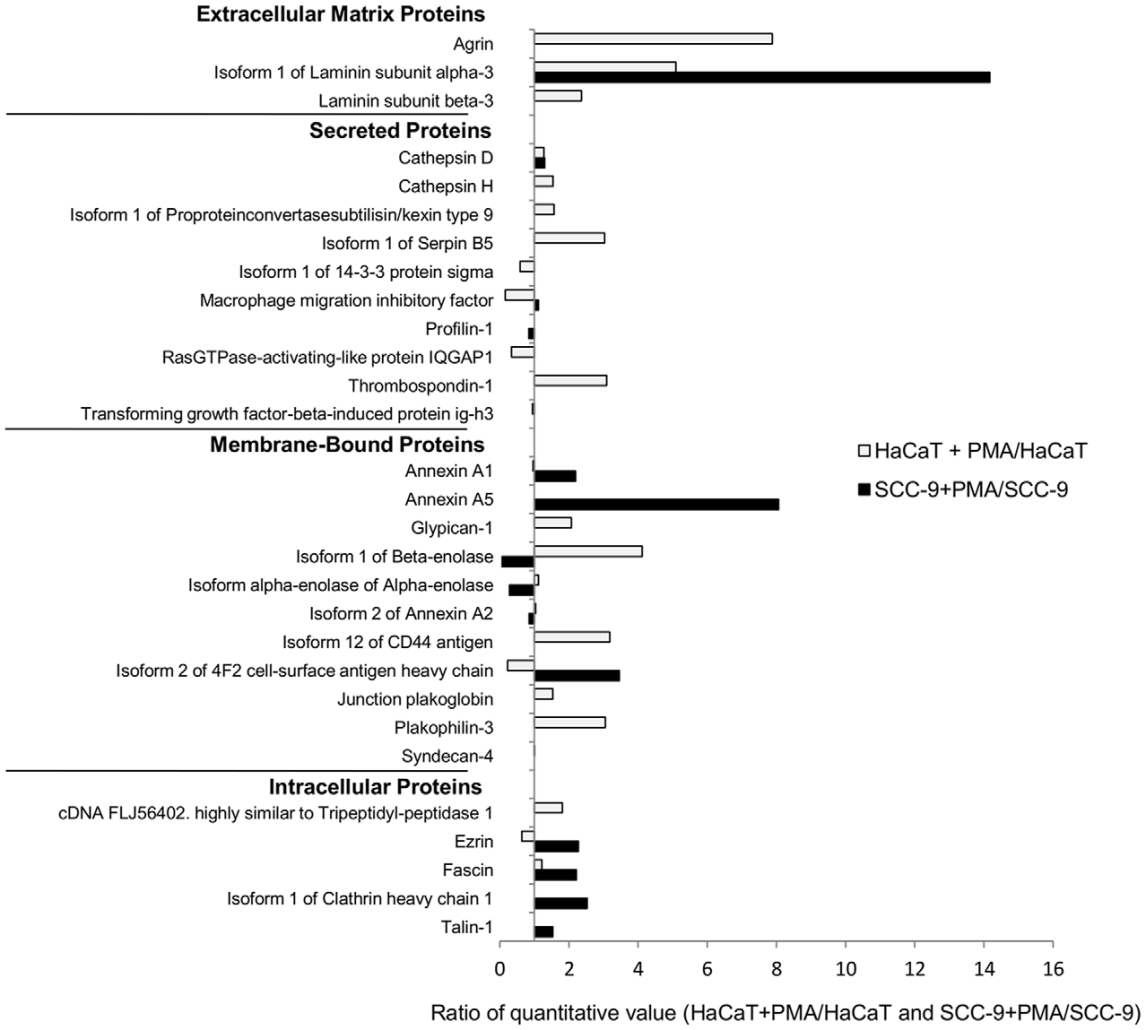
Identification of proteins in the conditioned media by LC-MS/MS according to the ratio of quantitative value (normalized spectral counts), as indicated in **Table 1**.

Fifty-three proteins were identified in the extracellular media and classified as extracellular matrix proteins, secreted proteins, membrane-bound proteins, and intracellular proteins that have a membrane projection. Differences between the cells either treated or not with PMA were observed, and based on the ratio of quantitative values, proteins with changes higher than 1.5-fold (i.e. >1.5 or <0.66) were considered as significantly regulated by PMA treatment (Table 1, Fig. 1).

**Table 1:** Identification of proteins in the conditioned media by LC-MS/MS according to the ratio of quantitative value.

Accession Number	Protein Identification	HaCat + PMA/HaCaT	SCC-9+ PMA/SCC-9	SCC-9/HaCaT	SCC-9+PMA/ HaCaT+PMA
	**Extracellular Matrix Proteins**				
IPI00374563	Agrin	7.88	absent	only in HaCaT	only in HaCat+PMA
IPI00022418	Isoform 1 of Fibronectin	absent	only in SCC+PMA	absent	only in SCC+PMA
IPI00003951	Isoform 1 of Laminin subunit alpha-3	5.09	14.17	0.46	1.29
IPI00015117	Isoform Long of Laminin subunit gamma-2	only in HaCaT + PMA	only in SCC+PMA	absent	0.70
IPI00783665	Laminin subunit alpha-5	only in HaCaT + PMA	absent	absent	only in HaCat+PMA
IPI00299404	Laminin subunit beta-3	2.36	Only in SCC+PMA	only in HaCaT	0.39
	**Secreted Proteins**				
IPI00011229	Cathepsin D	1.28	1.30	0.67	0.68
IPI00297487	Cathepsin H	1.54	absent	only in HaCaT	only in HaCat+PMA
IPI00021738	Collagenase 3 (MMP-13)	only in HaCaT + PMA	absent	absent	only in HaCat+PMA
IPI00023673	Galectin-3-binding protein	absent	only in SCC	only in SCC	absent
IPI00023728	Gamma-glutamyl hydrolase	only in HaCaT	absent	only in HaCaT	absent
IPI00008561	Interstitial collagenase	only in HaCaT + PMA	absent	absent	only in HaCat+PMA
IPI00026314	Isoform 1 of Gelsolin	only in HaCaT	absent	only in HaCaT	absent
IPI00291262	Isoform 1 of Clusterin	only in HaCaT	absent	only in HaCaT	absent
IPI00029658	Isoform 1 of EGF-containing fibulin-like extracellular matrix protein 1	only in HaCaT + PMA	absent	absent	only in HaCat+PMA
IPI00387168	Isoform 1 of Proprotein convertase subtilisin/kexin type 9	1.57	absent	only in HaCaT	only in HaCat+PMA
IPI00783625	Isoform 1 of Serpin B5	3.03	absent	only in HaCaT	only in HaCat+PMA
IPI00013890	Isoform 1 of 14-3-3 protein sigma	0.59	absent	only in HaCaT	only in HaCat+PMA
IPI00480121	Kallikrein-10	only in HaCaT + PMA	only in SCC+PMA	absent	10.43
IPI00293276	Macrophage migration inhibitory factor	0.16	1.12	0.86	5.97
IPI00216691	Profilin-1	absent	0.84	only in SCC	only in SCC+PMA
IPI00013895	Protein S100-A11	only in HaCaT	absent	only in HaCaT	absent
IPI00294879	Ran GTPase-activating protein 1	only in HaCaT	absent	only in HaCaT	absent
IPI00009342	Ras GTPase-activating-like protein IQGAP1	0.34	absent	only in HaCaT	only in HaCat+PMA
IPI00296099	Thrombospondin-1	3.09	only in SCC+PMA	only in HaCaT	0.20
IPI00018219	Transforming growth factor-beta-induced protein ig-h3	0.95	1.00	0.39	0.41
	**Membrane-bound proteins**				
IPI00218918	Annexin A1	0.96	2.19	0.85	1.93
IPI00024095	Annexin A3	absent	only in SCC	only in SCC	absent
IPI00329801	Annexin A5	absent	8.06	only in SCC	only in SCC+PMA
IPI00414320	cDNA FLJ55482. highly similar to Annexin A11	Only in HaCaT	absent	only in HaCaT	absent
IPI00015688	Glypican-1	2.07	absent	only in HaCaT	only in HaCat+PMA
IPI00010271	Isoform A of Ras-related C3 botulinum toxin substrate 1	absent	only in SCC	only in SCC	absent
IPI00218474	Isoform 1 of Beta-enolase	4.12	0.07	74.08	1.25
IPI00465248	Isoform alpha-enolase of Alpha-enolase	1.12	0.28	7.44	1.86
IPI00031030	Isoform 1 of Amyloid-like protein 2	only in HaCaT + PMA	absent	absent	only in HaCat+PMA
IPI00418169	Isoform 2 of Annexin A2	1.04	0.85	0.92	0.75
IPI00871158	Isoform 2 of Annexin A8-like protein 2	absent	only in SCC	only in SCC	absent
IPI00297160	Isoform 12 of CD44 antigen	3.18	only in SCC+PMA	only in HaCaT	1.31
IPI00027493	Isoform 2 of 4F2 cell-surface antigen heavy chain	0.23	3.46	1.58	24.32
IPI00554711	Junction plakoglobin	1.53	absent	only in HaCaT	only in HaCat+PMA
IPI00026952	Plakophilin-3	3.05	only in SCC	1.18	only in HaCat+PMA
IPI00002441	Syndecan-1	absent	only in SCC+PMA	absent	only in SCC+PMA
IPI00011564	Syndecan-4	1.01	only in SCC+PMA	only in HaCaT	8.18
	**Intracellular proteins**				
IPI00020599	Calreticulin	absent	only in SCC	only in SCC	absent
IPI00298237	cDNA FLJ56402. highly similar to Tripeptidyl-peptidase 1	1.81	absent	only in HaCaT	only in HaCat+PMA
IPI00843975	Ezrin	0.64	2.27	0.34	1.19
IPI00163187	Fascin	1.22	2.21	0.39	0.71
IPI00024067	Isoform 1 of Clathrin heavy chain 1	absent	2.53	only in SCC	only in SCC+PMA
IPI00014898	Isoform 1 of Plectin-1	absent	only in SCC	only in SCC	absent
IPI00291175	Isoform 1 of Vinculin	only in HaCaT + PMA	absent	absent	only in HaCat+PMA
IPI00219365	Moesin	only in HaCaT + PMA	only in SCC+PMA	absent	1.01
IPI00915869	Putative uncharacterized protein MDH1	absent	only in SCC	only in SCC	absent
IPI00298994	Talin-1	Only in HaCaT	1.53	24.56	only in SCC+PMA

The HaCaT and SCC-9 cells were treated with PMA for 24 h, the media were collected, the proteins were digested with trypsin and analyzed by LC-MS/MS. The data were submitted to Mascot search engine and the .dat files from Mascot output were analyzed in Scaffold Q+, which calculates the quantitative value by normalizing spectral counts across the experiments. The ratio of quantitative value of the PMA-stimulated cells/DMSO-treated cells for each protein is shown in the table. The proteins exclusively found in one condition are indicated as “only” and the proteins that are not present are indicated as “absent”.

Non-tumorigenic cells stimulated with PMA showed exclusively 14 up-regulated proteins, including agrin, laminin subunit alpha-5, cathepsin H, collagenase 3, interstitial collagenase, EGF-containing fibulin-like extracellular matrix protein 1, proprotein convertase subtilisin, serpin B5, amyloid-like protein 2, glypican-1, isoform 1 of beta-enolase, junction plakoglobin, plakophilin-3, tripeptidyl-peptidase 1 and vinculin. Tumorigenic cells also showed exclusively up-regulated proteins, such as fibronectin, annexin A1, annexin A5, 4F2 cell-surface antigen heavy chain, syndecan-1, syndecan-4, ezrin, fascin, clathrin and talin-1. However, there were commonly up-regulated proteins in response to PMA activation, namely laminin subunit alpha-3, laminin subunit beta-3, laminin subunit gamma-2, kallikrein-10, thrombospondin-1, CD44 antigen and moesin.

#### Peptidomic analysis reveals a potential candidate for cancer regulation

**Figure 2:**
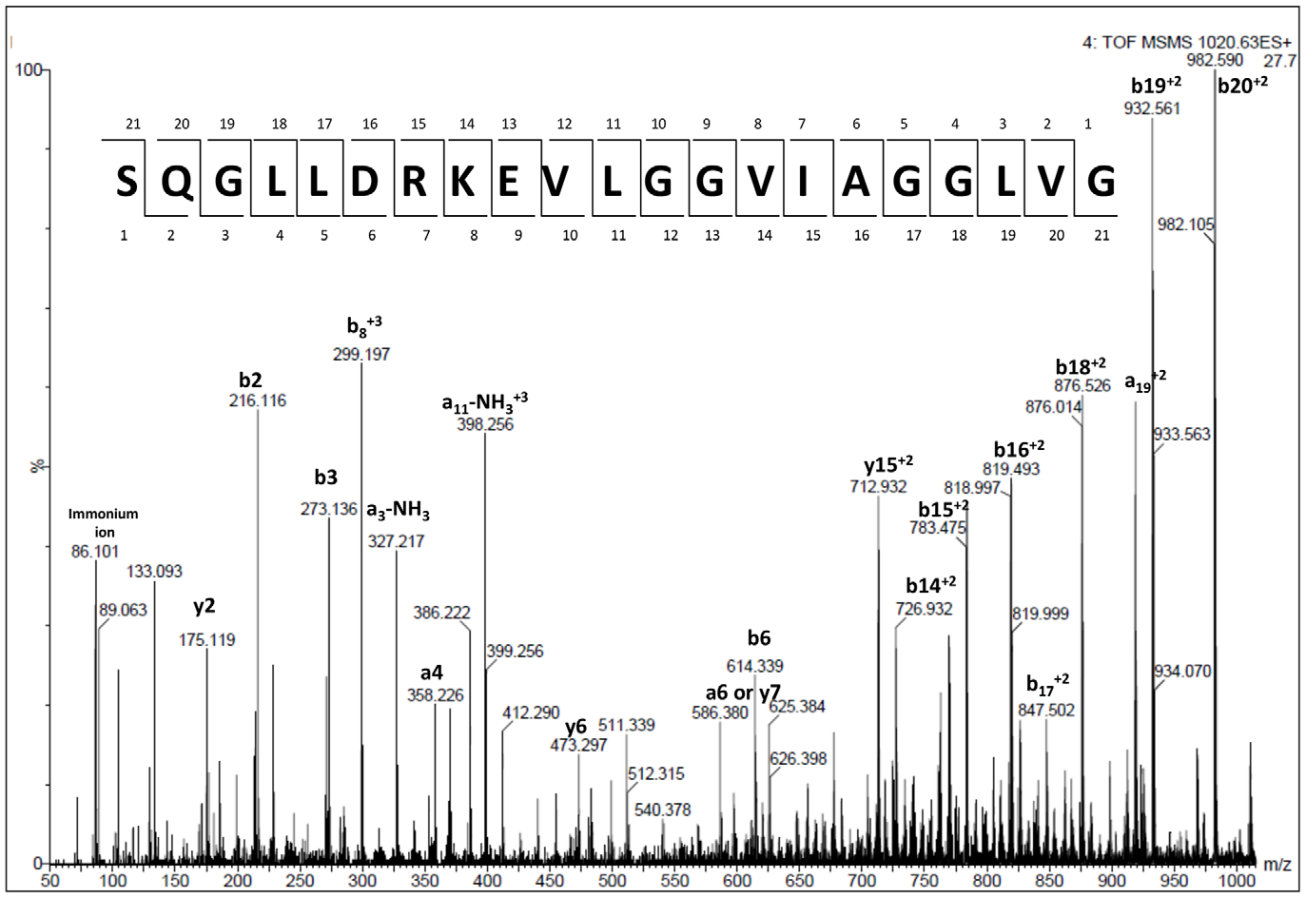
CID spectrum of a syndecan-1 peptide identified by LC-MS/MS. Endogenous peptides were identified by LC-MS/MS in the media after 24 h of PMA-treatment. The spectrum of syndecan-1 peptide (m/z 1019.5598, +2) was manually validated for b and y ion series.

The analysis of peptides in the media showed that the treatment with PMA induced the increase of syndecan-1 fragments (Table 2). Table 2 shows the number of unique peptides and number of spectral counts for each peptide sequence identified by LC-MS/MS. The syndecan-1-derived peptides increased mainly in tumorigenic cells stimulated with PMA (Mann-Whitney test, p = 0.0273). The higher number of spectral counts for syndecan-1-derived peptides was observed for the sequence SQGLLDRKEVLGGVIAGGLVG (SYN-1), for which the CID spectra were manually validated (Fig. 2).

**Table 2:** Identification of endogenous peptides in the conditioned media by LC-MS/MS according to the total number of unique peptides and spectral counts.

Accession number	Protein Identification	Experiment 1								Experiment 2							
		HaCaT		HaCaT+ PMA		SCC-9		SCC- 9+PMA		HaCaT		HaCaT+ PMA		SCC-9		SCC-9+ PMA	
		Run 1	Run 2	Run 1	Run 2	Run 1	Run 2	Run 1	Run 2	Run 1	Run 2	Run 1	Run 2	Run 1	Run 2	Run 1	Run 2
IPI00002441	**Syndecan-1**	**2/5**	**2/6**	**2/9**	**2/9**	**3/8**	**4/12**	**6/23**	**4/20**			**1/3**	**1/2**	**2/8**	**3/8**	**3/13**	**2/10**
	SQGLLDRKEVLGGVIAGGLVG	3	4	6	6	4	5	8	9			3	2	3	6	8	7
	SQGLLDRKEVLGGVIAGGLVGLI	2	2	3	3	3	4	5	4					5	1	3	3
	SQGLLDRKEVLGGVIA					1	2	5	5								
	LDRKEVLGGVIAGG							2							1		
	SQGLLDRKEVLGGVIAGG							1									
	RNQSPVDQGATGASQGLLDRKE							2	2							2	
	VLGGVIAGGLVG																
	DLHTPHTED						1										

The HaCaT and SCC-9 cells were treated with PMA for 24 h, the media were collected and the endogenous peptides were analyzed by LC-MS/MS. The total numbers of the unique peptides and the number of spectral counts are shown in bold and the number of spectral counts is shown for each sequence. There is a statistically significant difference in the number of spectral counts of syndecan-1 fragments between SCC-9 cells and SCC-9 cells treated with PMA (Mann-Whitney test, p = 0.0273).

### Analysis of the effect of PMA treatment on tumorigenic and non-tumorigenic cells

#### PMA induced cell migration in tumorigenic cells

**Figure 3:**
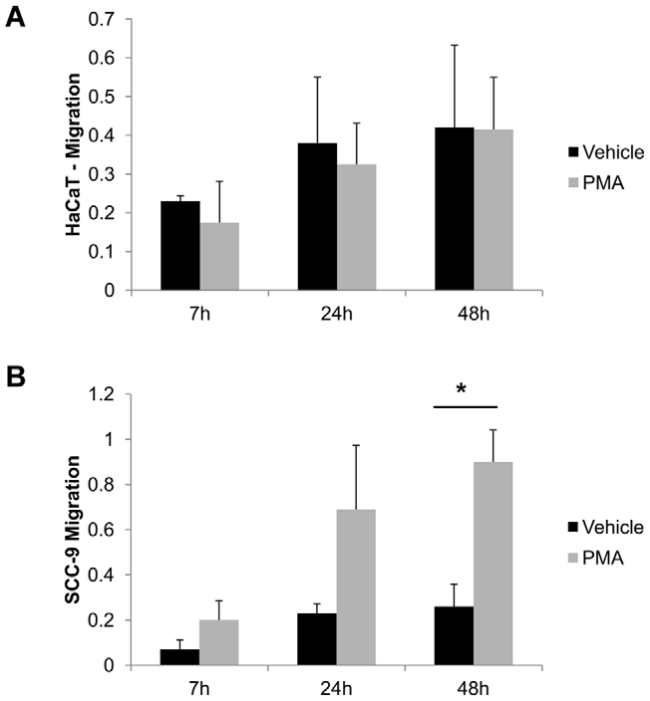
PMA increased migration only in SCC-9 cells by scratch assay. PMA and vehicle effect on the migration of HaCaT **(**A) and SCC-9 (B) cells after 7, 24 and 48 h. Cells grown in 12-well plates to confluence were scraped with p200 pipette tip. The closure was measure after 0, 7, 24 and 48 h and normalized to the control (vehicle: DMSO). Two independent experiments were performed with triplicates. Columns represent mean ± SD (n = 2) and * indicates p<0.01, compared to control (vehicle).

The effect of PMA on migration was evaluated by scratch assays (Fig. 3) and it was observed that PMA induced migration in SCC-9 cells, exclusively, upon 48 h-PMA treatment supplemented of 1% FBS (p** = **0.01, Students' *t*-test).

#### PMA promoted lower cell adhesion in HaCaT and SCC-9 cells

**Figure 4:**
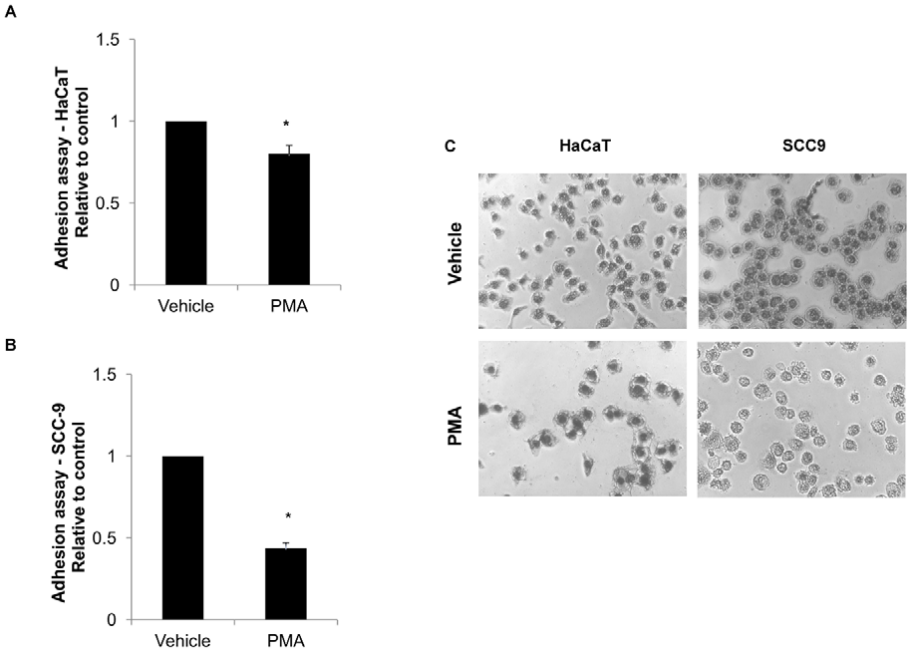
PMA promoted the loss of adhesion in Matrigel™ in HaCaT cells and SCC-9 cells. Adhesion assay showed that the cells stimulated by PMA decreased the ability to adhere to extracellular matrix proteins. HaCaT (A) and SCC-9 (B) cells had the ability to adhere in Matrigel™ diminished after 24 h of PMA treatment. Three independent experiments were performed with triplicates. Columns represent mean ± SD (n = 3) and * indicates p<0.01, normalized with the control (vehicle: DMSO). (C) Representative micrographs (magnification 40×) of adherent cells after PMA and vehicle (DMSO) treatments.

HaCaT (p = 0.002, Students' *t*-test) and SCC-9 (p<0.001, Students' *t*-test) cells showed reduced ability to adhere in Matrigel™ upon 24 h-PMA treatment, with a higher effect on SCC-9 cells (Fig. 4).

#### Gelatinase activity was increased with PMA stimuli

**Figure 5:**
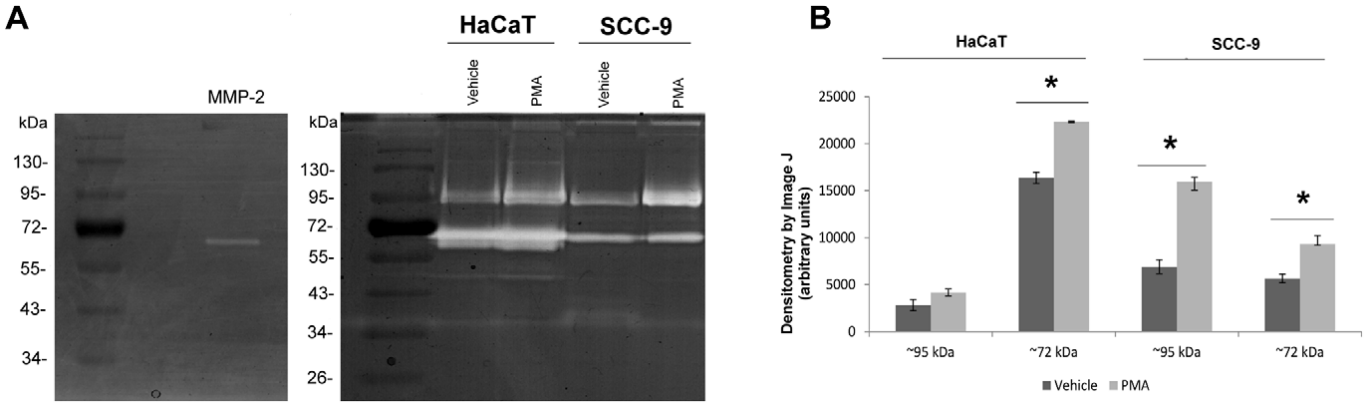
Gelatinase activity was increased with PMA stimuli. Representative 1D-zymography of conditioned media (12 µg of proteins) of HaCaT and SCC-9 cells treated or not with 50 ng/ml PMA two times with 12 h interval, collected after 24 h. The gelatinase activity of immunoprecipitated MMP-2 was used as positive control. Numbers on the left indicate the molecular mass marker mobility (A). The densitometry of clear areas showed the increase of activity after PMA treatment (B). Columns represent mean ± SD (n = 3) and * indicates p<0.05.

HaCaT and SCC-9 cells were treated with PMA and after 24 h the gelatinase activity was evaluated in the conditioned media. There was an increase of activity upon PMA treatment in both cell lines for ∼72 kDa gelatinase (p = 0.01 and p = 0.02 for HaCaT and SCC-9 cells, respectively, Students' *t*-test). The ∼95 kDa gelatinase showed higher activity upon PMA treatment only in SCC-9 cells (p = 0.04, Students' *t*-test). The activity of MMP-2 immunoprecipitated from the media confirmed the gelatinase activity (Fig. 5).

#### Membrane syndecan-1 decreased after 30 min and 24 h upon PMA treatment

**Figure 6:**
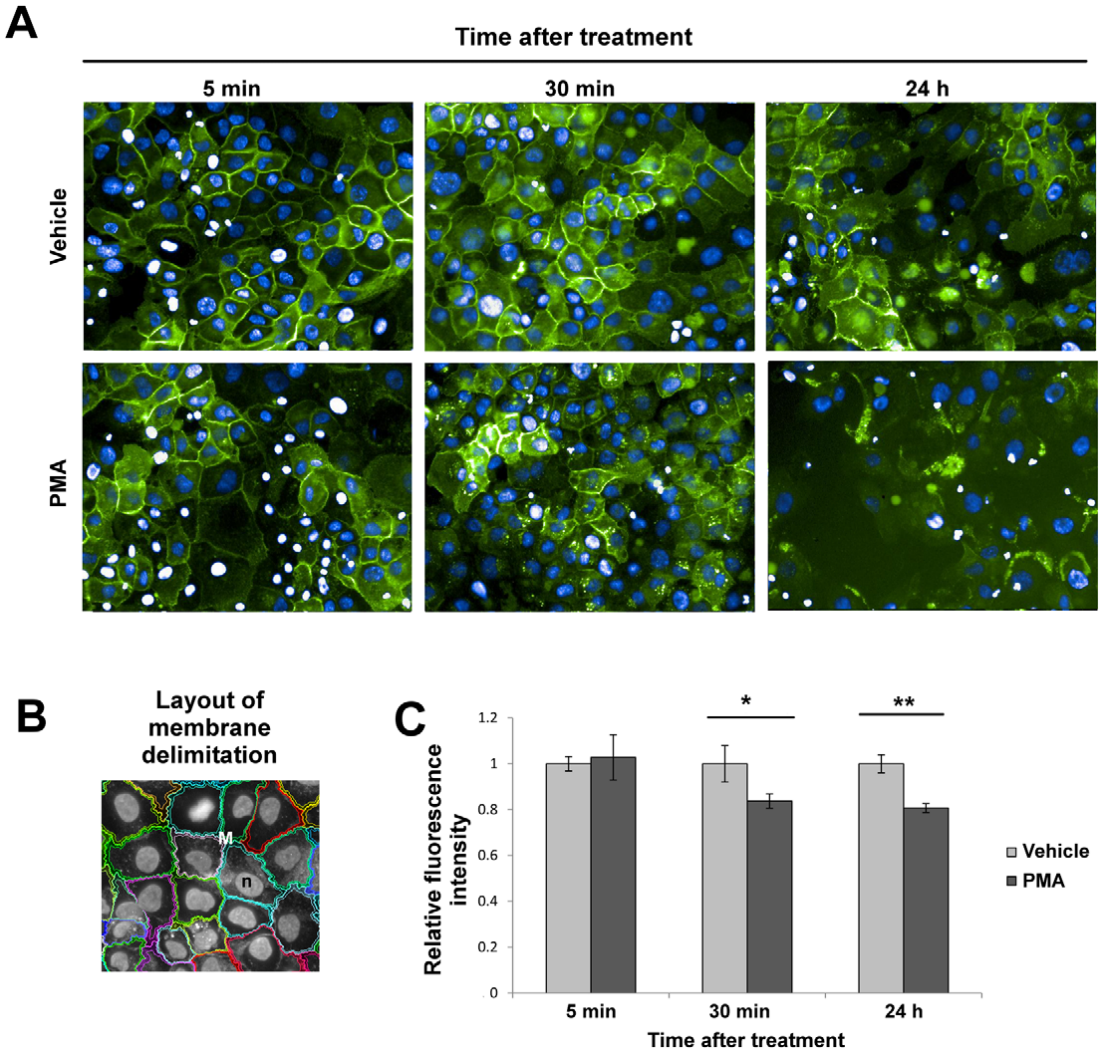
Loss of membrane-bound syndecan-1 localization after PMA treatment in SCC-9 cells. SCC-9 cells were treated with PMA for 5 min, 30 min and 24 h, fixed, and labeled for syndecan-1 with goat anti-syndecan-1 antibody. (A) Immunofluorescence images revealed diminished syndecan-1 in membrane localization after 30 min and 24 h of PMA treatment. Green: anti-goat antibody conjugated with Alexa Fluor 488. Blue: DAPI. (B) Example of masks used for quantification of syndecan-1 in the cell surface membrane. (C) Quantification performed by the Operetta high content image system showed loss of cell membrane of syndecan-1 after 30 min and 24 h of PMA treatment. Three independent experiments were performed. Columns represent mean ± SD (n = 3) and * and ** indicate p<0.05 and p<0.01, respectively.

The loss of membrane-bound syndecan-1 in SCC-9 cells was confirmed by immunofluorescence in a time-course experiment performed after 5 min, 30 min and 24 h upon PMA treatment (Fig. 6). The decrease of membrane-bound syndecan-1 was statistically significant after 30 min (Students' *t*-test, p = 0.02) and 24 h (Students' *t*-test, p = 0.001).

### Analysis of the effect of syndecan-1 synthetic peptide on tumorigenic and non-tumorigenic cells

#### Syndecan-1 synthetic peptide induced migration in tumorigenic and non-tumorigenic cells by scratch assay

**Figure 7:**
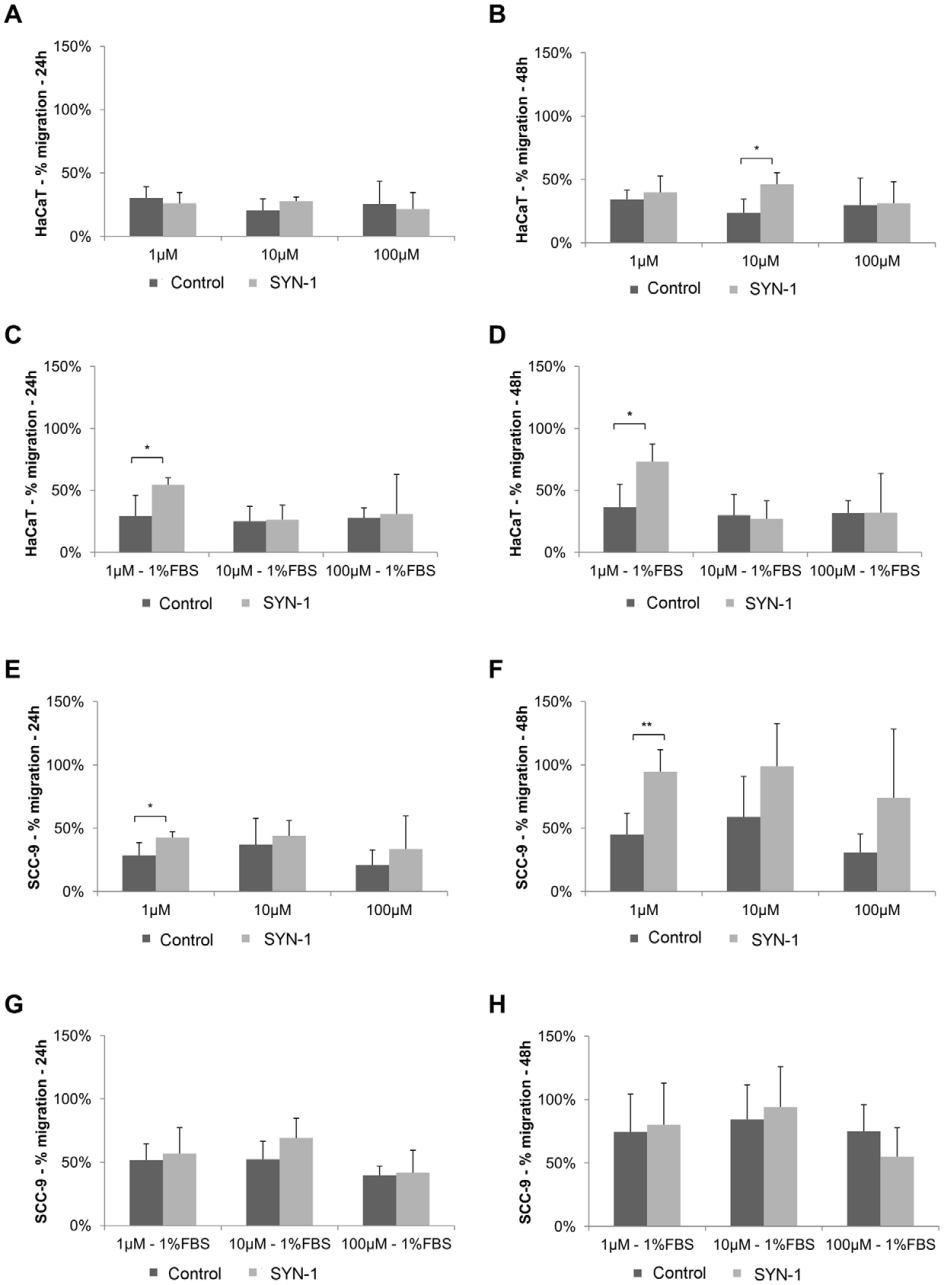
Synthetic syndecan-1-derived peptide promoted migration in HaCaT and SCC-9 cells by scratch assay. Migration of HaCaT and SCC-9 cells treated with synthetic syndecan-1 peptide (SYN-1) and its scrambled peptide (control) in the concentrations of 1 µM, 10 µM and 100 µM were evaluated by scratch assay. SYN-1 did not induce migration in HaCaT cells at 24 h (A), but 10 µM of SYN-1 promoted migration at 48 h in absence of FBS (B), 1 µM of SYN-1 induced migration in HaCaT cells at 24 h (C) and 48 h (D), both in the presence of 1% FBS. SCC-9 cell migration was statistically significant at the concentration of 1 µM of SYN-1 in the absence of FBS at 24 h (E) and 48 h (F), but it was not statistically significant when compared to the scrambled peptide in the presence of 1% FBS at 24 h (G) and 48 h (H). Three independent experiments were performed with duplicates. Columns represent mean ± SD (n = 3) and * and ** indicate p<0.05 and p<0.01, respectively.

The synthetic peptide of syndecan-1 (SYN-1) and its scrambled were evaluated at the concentrations of 1 µM, 10 µM and 100 µM by scratch assay. In HaCaT cells, 10 µM of SYN-1 peptide induced migration at 48 h in the absence of FBS (p = 0.02, Students' *t*-test) (Fig. 7B). In the presence of 1% FBS, it induced migration at the concentration of 1 µM, at 24 (p = 0.03, Students' *t*-test) and 48 h (p = 0.02, Students' *t*-test) (Fig. 7C and 7D). With respect to SCC-9 cells, SYN-1 peptide induced migration with statistical significance only at the concentration of 1 µM in the absence of FBS at 24 h (p = 0.04, Students' *t*-test) and 48 h (p = 0.01, Students' *t*-test) (Fig. 7E and 7F).

#### Syndecan-1 synthetic peptide induced migration in tumorigenic cells by transwell assay

**Figure 8:**
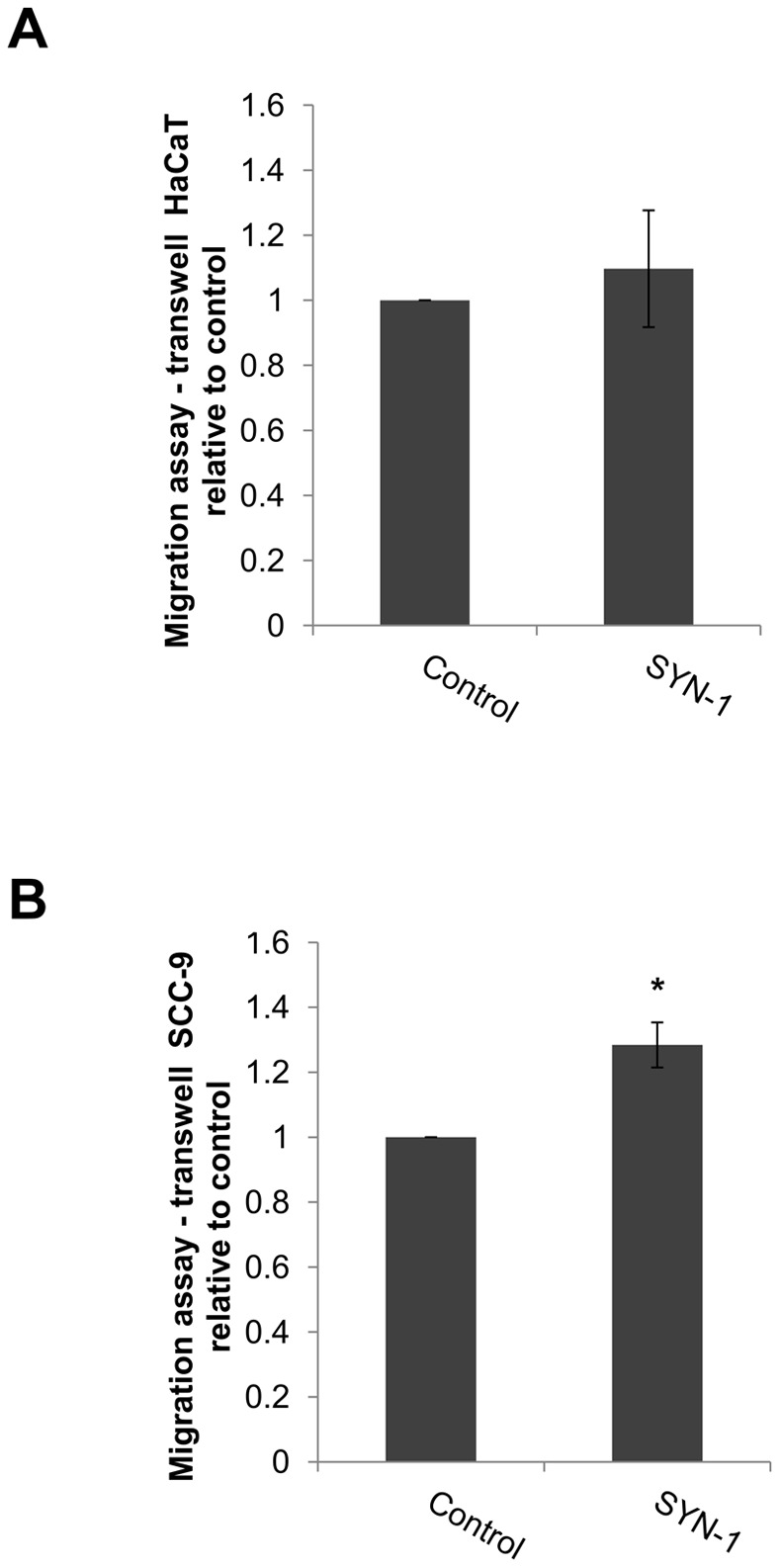
Synthetic syndecan-1-derived peptide promoted migration in SCC-9 cells by transwell migration assay. The migration of HaCaT and SCC-9 cells treated with synthetic syndecan-1-derived peptide (SYN-1) and its scrambled in a concentration of 10 µM in the absence of FBS was evaluated by transwell assay. The measurements were normalized with the control (scrambled peptide). SYN-1 did not induce migration in HaCaT cells (A). SCC-9 cell migration was statistically significant when compared to scrambled peptide (B). Three experiments were performed with triplicates. Columns represent mean ± SD (n = 3) and * indicate p<0.05.

The effect of SYN-1 peptide on migration was also evaluated by transwell assay at concentration of 10 µM in absence of FBS. The results showed an increase of migration only in SCC-9 cells (p = 0.02, Students' *t*-test) (Fig. 8B).

### Analysis of the effect of PMA treatment on tumorigenic and non-tumorigenic cells by qRT-PCR

#### mRNA levels of syndecan-1 did not change in a time-course experiment, but they showed higher expression in SCC-9 cells compared with HaCaT cells

**Figure 9:**
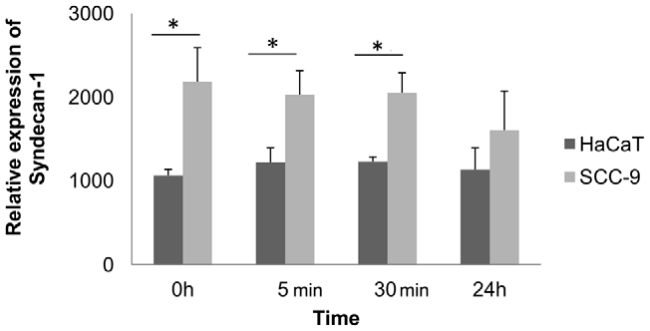
qRT-PCR analysis of syndecan-1 in HaCaT and SCC-9 cells treated with PMA at 0, 5 min, 30 min and 24 **h.** Relative mRNA expression levels of syndecan-1 were measured by the real-time quantitative PCR. The data were normalized with glyceraldehyde-3-phosphate dehydrogenase gene. One-way ANOVA analysis shows that PMA treatment did not increase the expression levels of syndecan-1 in each cell line in the time-course experiment. Student's *t*-test indicated differences in the expression level between the HaCaT and SCC-9 cells. Syndecan-1 showed higher expression in tumorigenic SCC-9 cells. Three independent experiments were performed with triplicates. Columns represent mean ± SD (n = 3) and * indicates p≤0.01.

One-way ANOVA analysis shows that PMA treatment did not change the mRNA expression levels of syndecan-1 in HaCaT and SCC-9 cells according to the time-course experiment (p>0.05). However, there were statistically significant differences in the expression levels of syndecan-1 between the cell lines at 0, 5 and 30 min upon PMA treatment (p = 0.009, 0.01 and 0.004, respectively) (Fig. 9).

## Discussion

Ectodomain shedding of cell surface proteins, secreted proteins and proteolysis-derived fragments are soluble protein candidates to contribute to a diverse extracellular milieu and subsequently certain biological events related to cancer [15], [16]. One percent of the extracellular ectodomains of membrane-anchored proteins are released from the cell surface by endogenous proteolytic cleavage [17], but the novel biological functions in which some of these molecules can be involved in cancer remain unclear. Here, we analyzed the secretome/sheddome of two epithelial cells lines in attempt to understand how they may affect tumor development. The non-tumorigenic (HaCaT) and tumorigenic (SCC-9) cells were stimulated with PMA that activates proteases and promotes protein shedding, secretion and proteolysis [2].

Differences between these cell lines, with or without stimulation by PMA, were observed in migration, adhesion as well as in gelatinase activity (Fig. 3, 4, 5, respectively). In agreement with these data, the secretomes/sheddomes were also different following stimulation with PMA (Fig. 1, Tables 1 and 2). From the proteomic analysis, seven proteins were up regulated by the effect of PMA treatment in both cell lines, while 14 and 10 proteins were exclusively found in HaCaT and SCC-9 cells, respectively (Fig. 1, Table 1). Consistent with these results, the secretome of human mammary epithelial (HMEC) cells, a non-tumorigenic cell line, also showed the increase of 36 extracellular proteins after PMA stimulation [2]. Similar to their findings, a significant number of PMA-stimulated proteins were related to proteolytic and extracellular matrix function. Interestingly, we found in the PMA-stimulated HaCaT cells, which are also non-tumorigenic, the increase of proteases expression, named cathepsin, kallikrein and collagenases. Besides, tumorigenic cells exclusively showed higher expression of membrane-bound proteins, such as syndecans. Furthermore, several PMA-stimulated proteins identified in the secretome of SCC-9 cells were also found in the secretome of other cancer cells [3], [4], [18], in which many of them represent potential biomarkers for cancer.

Among the identified proteins, heparan sulphate proteoglycans, such as syndecan-1, were found only in the media of stimulated SCC-9 cells (Table 1, Fig. 1). The increase of the soluble syndecan-1 in the media of stimulated SCC-9 cells was associated with the loss of syndecan-1 from the cell surface evaluated by immunofluorescence assay (Fig. 6). In fact, in a specific experiment to evaluate the syndecan-1 cleavage, previous study showed the increase of syndecan-1 cleavage in PMA-stimulated GAC myeloma cells compared with DMSO treatment, which was also enhanced or decreased, respectively, after Heparinase III and inhibitor (BB-94) treatments [19]. Interestingly, another large-scale secretome study to find cancer biomarkers also observed syndecan-1 in the extracellular media in three different types of cancer cell lines originated from oral cancer, colorectal carcinoma and lung cancer [18]. Furthermore, the loss of syndecan-1 from the membrane surface was previously correlated with carcinogenesis, suggesting that it could be a useful marker of malignant transformation or prognostic factor in oral squamous carcinoma [20], [21].

Interestingly, fragments of syndecan-1 were also found in higher abundance in stimulated SCC-9 cells (Table 2). We found seven peptides derived from the syndecan-1 and most of which have a leucine in the P1' position. Possible candidate proteinases responsible for such cleavage include ADAM-17 protease, which has valine or leucine residues as preferential sites at P1' position [22], and MMP-7 and MT1-MMP, which show a strong preference for leucine at P1' position [23], [24]. However, in this case it is not possible to predict which protease is responsible for the cleavage, since the media was collected 24 h after cell stimulation and more than one protease could had been involved in this process.

To support a functional role for soluble syndecan-1 fragments, we demonstrated that the synthetic syndecan-1 peptide (SYN-1) was able to induce cell migration in both cell lines, as observed by a scratch assay (Fig. 7), and also induced migration, by transwell assay, in tumorigenic cells, SCC-9 cells (Fig. 8). A recent study has demonstrated that both full-length and truncated syndecan-1 can modulate fibrosarcoma cell migration and adhesion, in which the extracellular domain is more important for promoting cell adhesion and the transmembrane and cytoplasmic domains in inhibiting cell migration [11]. In addition, a study using breast cancer cells observed that membrane-bound syndecan-1 increased cell proliferation, whereas soluble syndecan-1 increased cell invasion [25]. Another study showed that soluble syndecan-1 promoted growth of myeloma tumors [26]. Several studies have shown the involvement of syndecan-1 in cell migration and its regulation can occur through the regulation of the activities of integrins, including αvβ3, αvβ5, β4 and α2β1 [27]–[29] as well as by the cleavage of syndecan-1 by MMP-7, which could release the restrictions to migration [27]. However, here we showed the first example where a fragment of syndecan-1 was demonstrated to potentially be involved in cell migration. Based on previously investigations, it is possible that the soluble syndecan-1 also has an effect on carcinoma cells and it will be investigated in future studies.

The analysis of mRNA of syndecan-1 excluded the possibility that the increase of syndecan-1 in the media would be influenced by the increase of gene expression. We showed here that syndecan-1 mRNA expression levels did not change in a time-dependent manner (Fig. 9).

In summary, this study demonstrated that the repertoire of secreted, shed and degraded proteins in the extracellular milieu of PMA-stimulated non-tumorigenic HaCaT and tumorigenic SCC-9 cells could be involved in fundamental cell processes and reveals that a fragment of syndecan-1 was able to induce cell migration.

## Materials and Methods

### Cell Culture

The human OSCC cell line SCC-9 was obtained from American Type Culture Collection (ATCC, Manassas, VA, USA), and cultured as recommended. SCC-9 cells originated from human squamous carcinoma from the tongue. The HaCaT cells, an immortalized but not transformed epithelial cell line [30], was maintained in DMEM containing 10% fetal bovine serum (FBS) and antibiotics at 37°C in a 5% CO_2_ air atmosphere. HaCaT cells are human keratinocytes originated from skin.

### Mass Spectrometry Analysis: PMA Stimulation

The HaCaT and SCC-9 cells were cultured until 80% of confluence in 500-cm^2^ plates, washed with serum-free media and then stimulated two times, with 12 h interval, with 50 ng/ml PMA (Sigma) diluted in DMSO. The same concentration of DMSO was used as control in the time 0 and after 12 h. After 24 h, the media were collected and the proteins and peptides were analyzed as described below.

### Protein and Peptide extraction, Protein Digestion

The media were collected and the final concentration of 1 mM EDTA and 0.5 mM PMSF were added to the media. The protocol to obtain peptides and proteins was performed with few modifications [31]. Briefly, cell debris were eliminated by centrifugation at 4,000×*g* during 20 min at 4°C and the supernatants were heated in a water bath for 20 min at 80°C to inactivate proteases. After cooling, pH was adjusted to 2–3 by adding 10 M HCl to precipitate the proteins. After centrifugation at 10,000×*g* for 1 h at 4°C, the protein pellet was ressuspended in 200 mM ammonium bicarbonate and the peptides in the supernatant were concentrated in Sep-Pak® Vac tC18 cartridge 6cc/500 mg (Waters) and dried in a vaccum. The proteins in the extracellular media (50 µg) were treated with the final concentration of 4 M urea, following reduction, alkylation and digestion with trypsin (1∶50, w/w) [32].

### Mass spectrometry analysis

For protein analysis, an aliquot of 4.5 μl containing 15 μg of proteins of the resulting peptide mixture was evaluated as previously described [33] and for endogenous peptide analysis, we based on the intracellular protein concentration to inject similar concentration of peptides. Peptides (4.5 μl) were separated by C18 (100 μm×100 mm) RP-nanoUPLC (nanoAcquity, Waters) coupled with a Q-Tof Ultima mass spectrometer (Waters) with nanoelectrospray source at a flow rate of 0.6 μl/min. The gradient was 2–90% acetonitrile in 0.1% formic acid over 45 min for the digested proteins, and 60 min for endogenous peptides. The nanoelectrospray voltage was set to 3.5 kV, a cone voltage of 30 V and the source temperature was 100°C. The instrument was operated in the ‘top three’ mode, in which one MS spectrum is acquired followed by MS/MS of the top three most-intense peaks detected. After MS/MS fragmentation, the ion was placed on exclusion list for 60 s and for the analysis of endogenous cleavage peptides, a real time exclusion was used.

### Data Analysis

The spectra were acquired using software MassLynx v.4.1 and the raw data files were converted to a peak list format (mgf) without summing the scans by the software Mascot Distiller v.2.3.2.0, 2009 (Matrix Science Ldt.) allowing the label-free analysis, and searched against Human International Protein Database (IPI) v. 3.72 (86392 sequences, 35093930 residues; release date April, 2010) using Mascot engine v.2.3.01 (Matrix Science Ltd.), with carbamidomethylation as fixed modifications, oxidation of methionine as variable modification, one trypsin missed cleavage and a tolerance of 0.1 Da for both precursor and fragment ions. For the protein quantitation, the .dat files from Mascot output were analyzed in Scaffold Q+ (version 3_00_03, Proteome Software) and the quantitative value (normalized spectral counts) was obtained [34], [35]. For endogenous peptide identification, oxidation of methionine was set as variable modification, and a tolerance of 0.1 Da for both precursor and fragment ions. For label-free quantitation of endogenous peptides, the spectral counts [36] and the number of unique peptides were assessed. The peptide was considered as unique when it differs in at least 1 amino acid residue, covalently modified peptides, including N- or C-terminal elongation (i.e. missed cleavages) count as unique and different charge states of the same peptide and modifications were not count as unique. Only peptides with a minimum of five amino acid residues which showed significant threshold (p<0.05) in Mascot-based score were considered in the results. Two independent experiments were performed for proteins and peptides analysis. MS/MS spectrum of the most abundance endogenous peptide of syndecan-1 was manually validated for b and y ion series.

### Cell Migration Assay

The migration of HaCaT and SCC-9 cells was investigated through an in vitro monolayer assay. Cells grown in 12-well plates to confluence were scraped with a p200 pipette tip to create a cell-free area [37]. The cells were washed three times to remove cell debris. The same PMA treatment described above was performed for this assay (50 ng/ml PMA two times, with 12 h interval). The experiments were performed in serum-free media and 1% FBS supplemented media. The closure was evaluated after 0, 7, 24 and 48 h and analyzed by ImageJ software (http://rsb.info.nih.gov/ij/). Two independent experiments were performed in triplicates.

### Cell adhesion assay

The ability of HaCaT and SCC-9 cells to adhere to extracellular matrix proteins was evaluated in the adhesion assay [38]. First, the cells were plated in 100 mm dishes (Corning) at the density of 2×10^5^ cells for the PMA treatment (50 ng/ml, two times, with 12 h interval) and the another 96-well plate was coated with Matrigel™ (2 µg per well). After 24 h, these cells were trypsinised and seeded in this coated 96-well plate, previously washed three times with PBS and blocked with 3% BSA (bovine serum albumin) during 2 h in serum-free media supplemented with 3% BSA. The adhesion was evaluated for 1 h, and then the cells were washed and fixed with 10% formaldehyde. Briefly, the cells were stained with 1% toluidine blue containing 1% borax for 5 min. The dye was eluted using 1% SDS and the absorbance was measured at 620 nm. Three independent experiments were performed in triplicates.

### Zymography of conditioned media

For 1-D zymography, the proteins (12 μg) in the conditioned media collected after cell treatments with 50 ng/ml PMA two times with 12 h interval were submitted to 1-D electrophoresis on 12% SDS-polyacrylamide gels containing 1 mg/ml gelatin under nonreducing conditions, and gelatinolytic activity was performed as previously described [39]. We immunoprecipitated MMP-2, as positive control, from the conditioned media of HaCaT cells. Briefly, anti-MMP-2 antibody (1 μg) (Santa Cruz) was added to conditioned media containing 5 μg of proteins and incubated for 16 h at 4°C. The immunocomplexes were collected by incubating with 20 μl of protein-A Sepharose for 1 h at 4°C followed by three washed with 1% Tween in PBS. After centrifugation at 2,000×*g* for 2 min at 4°C, the beads were resuspended in sample buffer under nonreducing conditions as described before. Gels were stained with Coomassie blue and destained. Gelatin digestion was identified as clear bands against a blue background. Three independent experiments were performed.

### Immunofluorescence Assay

For immunofluorescence assays, SCC-9 cells were cultivated in a CellCarrier (Perkin Elmer) 384 well plate. After PMA stimulation as described above, the cells were analyzed after 5 min, 30 min and 24 h. The cells were washed with PBS, fixed with 4% formaldehyde for 10 min, washed again and permeabilized with 0.5% Triton X-100 for 10 min. The cells were blocked with blocking solution (PBS containing 0.2% Triton and 3% non-fat dry milk) for 30 min and then incubated with the goat anti-syndecan-1 antibody (R&D Systems) diluted in blocking solution for 1 h. Cells were washed with PBS and incubated with an anti-goat Alexa Fluor 488-conjugated antibody (Invitrogen), diluted in blocking solution for 1 h. Finally, the cells were washed with PBS, incubated with DAPI (4′,6-diamidino-2-phenylindole) solution for 10 min, washed again and analyzed by the Operetta high content image system (Perkin Elmer). All pictures were acquired with the same contrast and brightness parameters. For automatic fluorescence quantification in the Operetta platform, DAPI staining was used for cell counting. Syndecan-1 Alexa Fluor 488 staining, restricted to the cell membranes, was defined by the most appropriate mask. Three independent experiments were performed.

### Synthetic peptides

The peptide of syndecan-1 identified by MS and manually validated (H-SQGLLDRKEVLGGVIAGGLVG-OH), named SYN-1, and the scrambled control (H-IGVGGLRELVKQLGDLGGVSA-OH) were chosen for functional experiments. The peptide was synthesized (Proteimax, São Paulo, Brazil) with the same sequence identified by mass spectrometry.

### Cell Migration Assay with Syndecan-1-Derived Peptide

Migration of HaCaT and SCC-9 cells was performed as described before. Cells grown in 12-well plates to confluence were scraped with p200 pipette tip to create a cell-free area. The cells were washed three times with serum-free media to remove cell debris and incubated with serum-free media or 1% FBS containing the synthetic peptides SYN-1 and its scrambled in the concentrations of 1 µM, 10 µM and 100 µM. The migration was evaluated after 0, 24 and 48 h and analyzed by ImageJ software. Three independent experiments were performed with duplicates.

### Transwell migration assay with Syndecan-1-Derived Peptide

HaCaT and SCC-9 cells (7.5×10^4^ cells) were plated in the top chambers of 8 μm pore transwells (HTS Transwell-96 Well Plate, Corning) in the serum-free culture medium after a starvation period of 18 h. The cells were allowed to migrate towards serum-free medium supplemented with 10 µM SYN-1 peptide or its scrambled over a period of 6 h. At the end of the assay, cells at the top chamber were removed with a cotton swab and the cells at the bottom of the filter were fixed with 10% formaldehyde for 10 min, washed with PBS and stained with 1% toluidine blue solution in 1% borax for 5 min. The dye was eluted using 1% SDS and the absorbance was measured at 620 nm. Three independent experiments were performed in triplicates.

### Real-time quantitative PCR

In order to analyze the expression of syndecan-1, HaCaT and SCC-9 cells were cultured for 24 h in serum-free medium and treated with PMA during 5, 30 min and 24 h as described before. Total RNA was obtained using the TRIzol reagent (Invitrogen Corporation) and 2 µg of total RNA were used for retro-transcription using the First-Strand cDNA Synthesis Kit (GE Healthcare). Real-time quantitative PCR for syndecan-1 was performed using SYBR® Green PCR Master Mix (Applied Biosystems), and the dissociation curves were performed to confirm the specificity of products. The syndecan-1 forward primer was 5′- AGAAGAAGGACGAAGGCAGCTACT- 3′ and reverse primer was 5′- ATTCCTCCTGTTTGGTGGGCTTCT- 3′. The threshold cycles (CT) values of target gene were normalized relative to glyceraldehydes-3-phospate dehydrogenase gene, and relative expression ratios were calculated by the 2−ΔΔ Ct method. Three-independent experiments were performed with duplicates.

### Statistical analysis

For the functional experiments, the assumptions of adherence of the errors to the Gaussian distribution were tested, for all response variables, using the Shapiro-Wilk Test. The GLIMMIX procedure of the SAS System (SAS Institute Inc. The SAS System, release 9.12. SAS Institute Inc., Cary:NC, 2008) were used to calculate the analysis of variance followed Student's *t*-test or Tukey test. For the analysis of mRNA expression of syndecan-1, the analysis of variance was followed by Tukey test. For the statistical analysis of syndecan-1 fragments, it was used Mann-Whitney test to compare the effect of PMA on HaCaT and SCC-9 cells. The level of significance was stated at 0.05 in all statistical tests applied.
